# An Essay on the Therapeutic Virtues of the Euonymus Atropurpureus or Wahoo

**Published:** 1846-04

**Authors:** Ellwood Andrews

**Affiliations:** Attica, Indiana


*An Essay on the Therapeutic Virtues of the Euonymus Atropurpureus, or Wahoo.* Written for the Degree of Doctor of Medicine in the "Rush Medical College." By Ellwood Andrew, of Attica, Indiana. Presented Feb. 2, 1846.

Euonymus Atropurpureus.
Spindle Tree.
Burning Bush.
Wahoo. Radicis cortex. The bark of the root.

Class, Petandria. Order, Monogynia.

Flowers axilate in small open umbels, four equal stamens, anthers two lobed, one pistil, four equal petals, calyx four equal sepals; the petals and sepals same color, dark red. Berries of a bright scarlet color, fleshy, four lobed, at right angles; each lobe containing one seed in capsule. Branches opposite and four angled. Leaves opposite on short foot stalks, ovate acuminate, veined and finely serrated, color, dark green on the upper surface, lighter beneath.

The wahoo is a beautiful and ornamental shrub, attaining from six to twelve feet in height, and may be found throughout the Northern and Middle, and perhaps over the whole of the United States. It delights in a rich and loose soil; and is generally found on the margins of running streams, on hill sides, &c. It flowers in June, and the fruit matures in September.

This shrub is liable to be mistaken for the young grey ash, which it much resembles in its general appearance. It may, however, be easily distinguished in autumn, by the fruit remaining on the tree long after the surrounding foliage has disappeared. This shrub is covered with a thin, and for the most part, smooth epidermis, which is of a dark ash color, gradually becoming green, however, on the younger branches. The roots, which are small and numerous, are covered with a bark of a pale cineritious color. The taste of the bark of the root is a pleasant bitter, slightly pungent. It possesses a faint odor. Both odor and taste much resemble that of Ipecacuanha. The bark is tough and difficult to pulverise. Water and alcohol extract its virtues. There are diversities of opinion among botanists in reference to the proper botanical name of the wahoo. Some regard it to be the *ulmus alata*, which also bears the specific name of wahoo. I am satisfied, however, that it does not belong to that family, and I have chosen, in accordance with the views of Dr. Kennicott, and other able botanists, whom I have consulted, to consider it as the E. Atropurpureus in preference to the E. Americanus, as it answers more nearly to the description of the former than the latter variety.

When this substance was first known as a remedy, it is impossible at this time to determine. It has, however, long enjoyed a reputation as a valuable expectorant in pulmonary diseases. For this purpose a variety of preparations of it have appeared from time to time, usually in the form of syrups.

As a Tonic, it enters largely into the various popular compounds, known as bitters, and as such, used in various conditions of the system; such for example, as rheumatism, indigestion, want of appetite, &c., and is extensively used during convalescence from autumnal intermittents. It is frequently exhibited in the form of decoction, as a diaphoretic and diuretic in obstruction of the urinary organs. It is an exceedingly popular remedy as a cathartic in a great number of diseases, where a remedy of this class is indicated. In autumnal fevers this substance is frequently administered by the country people, without regard to the form of fever or the stage of the disease, and generally with success, at least in such cases as may be cured by active cathartics.

The frequent exhibition of this substance by the community, and the benefits that uniformly appeared to result from its use, have gradually brought it to the notice of physicians, many of whom have been induced to prescribe it in various forms of disease. From the opinion of a number of physicians, who are in the habit of prescribing the wahoo, no doubt can be entertained of its virtues as a remedy, and of its claims to a place in the Materia Medica.

The physiological action of the wahoo appears to be twofold, which depends upon its tonic and cathartic properties. As a tonic, its action resembles that of the vegetable tonics in general; producing, when administered in small doses, an increased cutaneous and pulmonary secretion, and giving tone and vigor to the system generally, without manifesting any marked stimulating properties. When thus administered, it excites, in a peculiar manner, the secretion from the urinary organs. There are few remedies that possess this quality in a higher degree. Should the dose be increased beyond what is requisite to produce a diuretic effect, it acts as a mild cathartic or laxative, leaving the bowels in a soluble condition.

In no instance, however, is its action more distinctly manifested than when given in full doses. When thus administered, it acts as a powerful hydrogogue cathartic, producing copious fluid evacuations, which frequently continue for twenty-four or thirty-six hours after its administration. Notwithstanding its powerful hydrogogue effects, it does not produce any drastic effects; its action being attended with little or no pain. Another peculiarity of this substance is, that when its use is continued for a length of time, it does not, like most remedies of this class, lose its hydrogogue effects. On the contrary, it appears to be as prompt and efficient in its action as when first administered.

From the foregoing remarks, it will be seen that the wahoo possesses medical virtues of no ordinary character, and that it strongly recommends itself to the attention of the profession. From the brief views that have been given of its mode of action upon the system, it will not be difficult to determine when its administration may be indicated, or when it may be prescribed with advantage.

In plethoric habits, when there is a determination of blood to the head, the wahoo may be given in cathartic doses, and will be found to dispel all the unpleasant symptoms.

The catarrhal affections of the air passages, which are of so frequent occurrence in our climate, may frequently be promptly relieved by the free use of this remedy. In chronic affections of the lungs, attended with cough, this article has long been used and is frequently prescribed with advantage.

A number of instances have fallen under my own observation, where this remedy has been used in chronic coughs from various causes, used with more benefit than any other In these cases it would seem to possess some specific action over the mucous membrane of the lungs; throwing off the disease by promoting a healthy secretion from that surface.

It has been found of much service in the treatment of various forms of disease, depending on a miasmatic influence. In rheumatic inflammations, depending upon this cause, it may be given with decided advantage. Here, as well as in all other cases, where there is much inflammatory excitement, bloodletting, either general or local, should be premised.

Of the multifarious forms of disease in which this substance has been administered, there are none in which its beneficial effects are more strikingly exhibited, than in dropsical effusions. In cases of this kind, where the amount of fluid is so enormous or the effusion is so rapid, that remedies ordinarily employed, are wholly inadequate to the task of removal, or inadmissable from the great debility they produce, or from their continued use become inert, the wahoo would seem to be particularly indicated; filling as far as it is possible for one remedy to do, all the indications.

From the testimony of a number of physicians, who have had occasion to use this article in these as well as other forms of disease, the following remarks from Drs. Evans and Buell, of Indiana, are well worthy of our attention, combining, as they do, in a few words the opinion of these gentlemen, who have had much experience in its use. Dr. Buell remarks, after speaking of its tonic and cathartic virtues, "in witnessing these properties of the wahoo, I did not hesitate to prescribe it in the various forms of dropsy that came under my notice, in conjunction with such other remedies as each particular case seemed to require, and I have had much reason to be pleased with its effects, and at no time to regret its administration." He considers it particularly indicated in effusions in the cellular membranes, and further remarks, "that in these cases, it will rarely disappoint our most sanguine expectations." In hydro-thorax and ascites, he cannot speak with as much confidence, having failed to perform a *cure* in both these forms of disease, but as they are frequently dependant upon a lesion of some important organ, in which all other remedies fail in a cure, he therefore does not consider them as a test of the therapeutic virtues of the wahoo. He only reports one case in which this article was administered without *any* benefit, and this was a case of anasarca, resulting as the sequelæ of scarletina, which had been long neglected. The presence of febrile symptoms, he does not deem an objection to the use of this article in cathartic doses, more particularly should depletion first be employed. He is confirmed in this opinion by the fact before alluded to, that it is frequently used by the country people, as a domestic remedy in the cure of autumnal fevers, and generally with the happiest results in those cases that demand active purging. The views of Dr. Evans strongly corroborate the remarks of Dr. Buell. Dr. Evans remarks, "from the observations that he has been enabled to make, he is decidedly in favor of its use in most cases of dropsy, where an evacuant is indicated." In speaking of its physiological action, he continues, "one drachm of the bark of the root, gathered in the fall or winter, (which is the proper time) infused in six ounces of water, will, if given at four or six equal doses every two hours, have a decided diuretic effect, unless it should operate on the bowels. In doses of twice or thrice this amount, it acts as an energetic hydrogogue cathartic, producing a number of evacuations. It is a remarkable property of this medicine, that the frequent exhibition of it as a cathartic, does not, as is the case with most, if not all the remedies of this class, destroy its hydrogogue effects. I have given it every other day for two weeks, and the last dose seemed to produce the same effects as the first." He also speaks of its tonic properties. "From these properties, I regard it as one of the very best remedies that can be given in dropsies where the system is much prostrated, and the accumulation of the watery fluids rapid. In one case of ascites and hydrops pericardii, with general anasarca of several months standing, which evidently was attended with a miasmatic influence, originating in an attack of remittent fever, and attended with enlargement of the spleen, I had reason to attribute the cure to the effects of wahoo, given every other day in cathartic doses, in conjunction with quinine and the application of blisters over the region of the spleen."

Another and severe case of ascites, hydro thorax and general anasarca, which occurred in an individual of a chlorotic habit, came under the notice of Dr. Hays, in which the specific action of this remedy in this form of disease, was most strikingly exhibited. The patient, a young lady in whom the catamenia had never been instituted, had long been afflicted with rheumatic inflammation of almost the whole fibrous tissue of the body, which gave way to, or rather was the cause of the dropsical effusion. The system was much prostrated, the accumulation of fluids rapid, and so abundant as to seriously impede respiration; causing frequent fits of suffocation, which threatened the life of the patient. In this case the wahoo was the only remedy that appeared sufficiently powerful for the re-removal of the fluids, without at the same time impairing the strength of the patient, and notwithstanding the patient died, as might have been expected, no doubt could be entertained that life had been prolonged and rendered more endurable by the free use of this remedy; the dropsical effusions having been entirely removed long before death, and the patient dying from other causes. It has been seen elsewhere, that this article is particularly adapted to dropsical effusions of the cellular membranes. There are a great number of diseases, which produce serous effusions into this tissue of the body; in a majority of cases, perhaps, the effusion is produced from the same cause, namely: that of atony or debility of the system. A disease in which this infiltration frequently obtains, is scarlatina. Again, in badly treated cases of autumnal fevers, or in cases that have not been treated at all, we not unfrequently find in addition to the anasarca which is present, a derangement of the bowels, want of appetite, &c. In all these cases a careful examination into the history and cause of the disease, and a just discrimination in the selection of remedies, will at once determine whether this remedy is applicable to the case, and in many cases it will doubtless prove of much service.

The foregoing are a few of the many cases, that might be adduced, where the wahoo has been used in dropsical effusions. Its value, as a remedy in these cases, appears to depend on its power of exciting the secretions from the alimentary canal; and through this means causing the rapid absorption of fluids from other parts of the body. In many cases of serous effusions, the removal of the fluids is the only indication to be fulfilled. In such cases this remedy may be considered of primary importance. Other cases again occur, in which the dropsy is only a symptom of the disease, the effusion depending upon a lesion of some organ, over which this remedy has no control. In such cases this article should be considered only as an adjuvant, and be used in conjunction with such other remedies as the primary disease might seem to demand. Cases indeed frequently occur in which the wahoo is wholly inadmissible, requiring remedies of the most opposite character. In those, for example, in which the disease is produced by the long continued and excessive use of spirituous liquors, where there is intestinal disease, &c., this remedy would be of doubtful efficacy, and should be prescribed with caution.

It has been heretofore remarked that this remedy had been used successfully in the treatment of autumnal fevers. I am not aware, however, in what particular form of fever its use has been of most benefit; neither am I aware of any physician who relies on it in the commencement of these diseases. Cases are not wanting, however, where its empyrical use has been of decided benefit in the commencement of remittents and intermittents. When prescribed in the commencement of these diseases, it should be exhibited with caution and be premised with blood-letting, or such other remedies as the case might seem to require. Cases have occurred where its too liberal use has produced hyper-catharsis. In the latter stages of these diseases, where a tonic and aperient is indicated, it may be resorted to in many cases with decided advantage; this point, however, has been sufficiently dwelt upon elsewhere.

The proper time for collecting the root of the wahoo is late in autumn or in the winter. No particular directions are necessary for its preparation, farther than to observe that it should be collected at a proper time, cleansed by washing, and the bark removed from the root and dried. The drying process may be conducted either by the heat of the sun in the open air, or by the aid of a moderate heat from a stove.

The mode of administering this substance may vary according to the indication to be fulfilled, or the convenience of administration. When administered for its tonic properties alone, the tincture may be used. This may be prepared of any strength that may be desired. From one to two ounces of the bark may be added to a pint of proof spirits, and permitted to digest for a sufficient length of time, which will afford a tincture of sufficient strength for ordinary purposes. Of this from fʒi to f℥ss may be taken at once, and repeated as often as occasion may require.

The infusion is perhaps the best form in which to administer it with a view to obtain its diaphoretic and diuretic effects. To prepare this, an ounce of the bark may be infused into a pint of boiling water, of which from f℥i to f℥iii may be given at stated periods.

The decoction is the form which is usually given as a cathartic, and is prepared by adding from one to two ounces of the bark to a pint of boiling water, and continuing the heat until the strength of the root is extracted. Of this from f℥ss to f℥ii may be taken, and repeated at intervals of from two to four hours, according to the urgency of the case, until its full cathartic effects are produced. Seldom, however, will it be necessary to repeat more than the second or third dose. The only unpleasant symptoms that I have known to arise from its use, has been hyper-catharsis when it was exhibited in too large doses. Some care, therefore, should be taken to prevent this occurrence. Should it occur, however, the remedies ordinarily employed in such cases, will be available. Another manner in which this substance may be given is in the form of an extract, which has recently been prepared by G. W. Carpenter, of Philadelphia, and which is much extolled for its remedial virtues and facilities of administration. I have, however, been unable to procure a specimen for examination, and am unable to speak of its physical properties.

